# Temporal analysis and spatial distribution of acquired syphilis in the state of Mato Grosso, Brazil, 2010-2021: an ecological study

**DOI:** 10.1590/S2237-96222024V33E2023398.EN

**Published:** 2024-01-22

**Authors:** Susi Astolfo, Amanda Cristina de Souza Andrade, Ruth Terezinha Kehrig

**Affiliations:** 1 Superintendência Estadual do Ministério da Saúde, Serviço de Articulação Interfederativa e Participativa, Cuiabá, MT, Brazil; 2 Universidade Federal de Mato Grosso, Instituto de Saúde Coletiva, Cuiabá, MT, Brazil

**Keywords:** Sexually Transmitted Diseases, Syphilis, Geographic Mapping, Enfermedades de Transmisión Sexual, Sífilis, Mapeo Geográfico, Doenças Sexualmente Transmissíveis, Sífilis, Mapeamento Geográfico

## Abstract

**Objective:**

To analyze the temporal trend and the spatial distribution of acquired syphilis in Mato Grosso, Brazil, between 2010 and 2021.

**Methods:**

This was an ecological study using notifications of acquired syphilis held on the Notifiable Health Conditions Information System. Detection rates were calculated by health macro-region and three-year periods (2010-2012, 2013-2015, 2016-2018, 2019-2021). The jointpoint method was used to calculate annual percentage change (APC). Thematic maps of Bayesian rates were built and distribution was analyzed using Local Moran.

**Results:**

The detection rate increased from 16.2 per 100,000 inhabitants in the first three-year period (2010-2012) to 70.0 in the last three-year period (2019-2021). The Central-North macro-region had the highest rate in the last three years (94.3/100,000 inhab.), while the highest upward trend occurred in the Central-Northwest macro-region, from 2013 to 2018 (APC = 50.2; 95%CI 26.3;78.6). There was an increase in Bayesian rates in most municipalities.

**Conclusion:**

There was a trend towards an increase in acquired syphilis, especially in the last two three-year periods.

## Study contributions

**Main results:**

The syphilis detection rate increased from the first three-year period (2010-2012) to the last period (2019-2021), being the highest in the Central-North macro-region in the last three-year period. The greatest variation occurred in the Central-Northwest macro-region (2013-2018). Bayesian rates increased in most municipalities.

**Implications for services:**

Promotion, prevention and treatment actions must be directed to appropriate locations, contributing to improving disease control indicators and making better use of available material resources.

**Perspectives:**

It is expected that the results of the study will contribute to syphilis control strategies, by researchers, health professionals and the population, as well as contributing to greater prevention effectiveness, including records for monitoring cases in the continuum of care.

## Resumen

**Objetivo:**

Analizar la tendencia temporal y la distribución espacial de la sífilis adquirida en Mato Grosso, de 2010 a 2021.

**Métodos:**

Estudio ecológico utilizando informes de sífilis adquirida del Sistema de Información de Enfermedades de Declaración Obligatoria - Sinan. Las tasas de detección se calcularon por macrorregión de salud y trienios (2010-2012, 2013-2015, 2016-2018, 2019-2021). Se utilizó el método jointpoint para calcular el cambio porcentual anual (CPA). Se construyeron mapas temáticos de tasas bayesianas y se analizó la distribución por Moran Local.

**Resultados:**

La tasa de detección aumentó de 16,2 por 100.000 en el primer trienio (2010-2012) a 70,0 en el último trienio (2019-2021). La macrorregión Centro-Norte presentó la tasa más alta en los últimos tres años (94,3/100.000 hab.), mientras que la mayor tendencia al alza se presentó en la macrorregión Centro-Noroeste, de 2013 a 2018 (CPA = 50,2; IC_95%_ 26,3; 78,6). Hubo un aumento en las tasas bayesianas en la mayoría de los municipios.

**Conclusión:**

Hubo una tendencia al aumento de la sífilis adquirida, especialmente en los dos últimos trienios.

## Resumo

**Objetivo:**

Analisar a tendência temporal e a distribuição espacial da sífilis adquirida em Mato Grosso, de 2010 a 2021.

**Métodos:**

Estudo ecológico utilizando notificações de sífilis adquirida do Sistema de Informação de Agravos de Notificação. As taxas de detecção foram calculadas por macrorregiões de saúde e triênios (2010-2012, 2013-2015, 2016-2018, 2019-2021). O método *jointpoint* foi utilizado no cálculo da variação percentual anual (VPA). Foram construídos mapas temáticos das taxas bayesianas e a distribuição analisada por Moran local.

**Resultados:**

A taxa de detecção passou de 16,2/100 mil habitantes, no primeiro triênio (2010-2012), para 70, no último triênio (2019-2021). A macrorregião Centro-Norte apresentou a maior taxa no último triênio (94,3/100 mil hab.), enquanto a maior tendência de aumento ocorreu na macrorregião Centro-Noroeste, de 2013 a 2018 (VPA = 50,2; IC_95%_ 26,3;78,6). As taxas bayesianas aumentaram na maioria dos municípios.

**Conclusão:**

Houve tendência de aumento da sífilis adquirida, principalmente nos dois últimos triênios.

## INTRODUCTION

Acquired syphilis refers to all forms of syphilis, with the exception of congenital syphilis, the approach to which is different from the other forms. Although syphilis in pregnant women is an acquired form of the disease, it is reported separately. The health information systems used to notify syphilis in Brazil are the Notifiable Health Conditions Information System (*Sistema de Informação de Agravos de Notificação* - SINAN) and the e-SUS/Health Surveillance System (*e-SUS/Vigilância em Saúde*).

Syphilis is a sexually transmitted bacterial infection (STI) that has become an epidemic, in view of its growth as seen in most middle-income countries, including Brazil.^1^ According to the World Health Organization (WHO), 7.1 million adults contracted syphilis in 2020.^2^

The increase in syphilis cases in Brazil has been recorded in the Ministry of Health Epidemiological Bulletin on Syphilis, which reported an increase in incidence from 2 cases per 100,000 inhabitants in 2010, to 9,3 cases per 100,000 inhabitants in 2011.^3^ This upward trend has been attributed to factors such as expansion of testing, especially rapid testing; reduction in condom use; primary health care worker resistance to administering penicillin (which can be justified by the fear of risk of allergic reactions to it); worldwide shortages of penicillin; and improvement of the syphilis surveillance system.^4^

In view of the severity of the epidemiological scenario of syphilis in Brazil, in 2016 the Ministry of Health declared syphilis to be an epidemic. This required strategic actions on the part of health authorities, with the aim of reducing its occurrence in the country’s states.^5^ In 2017, with the aim of reducing syphilis transmission at the national level, in partnership with the *Universidade Federal do Rio Grande do Norte*, the Ministry of Health launched the Interfederative Project for Rapid Response to Syphilis in Healthcare Networks (*Projeto Interfederativo de Resposta Rápida à Sífilis nas Redes de Atenção*).^5^ In 2019, the Clinical Protocol and Therapeutic Guidelines for Comprehensive Care for People with STIs (*Protocolo Clínico e Diretrizes Terapêuticas para Atenção Integral às Pessoas com IST* - PCDT) brought, as an innovation, the development of a clinical decision algorithm for syphilis case management, with recommendations for testing, diagnosing, treating, reporting and follow-up of syphilis cases.^1^ ^),(^ ^4^

Several published studies show the evolution of congenital syphilis and acquired syphilis in pregnant women nationally, in the Brazilian states and in its Federal District.^6^ ^)-(^ ^10^ ^)^ Notwithstanding, epidemiological studies analyzing the occurrence of acquired syphilis are scarce.^11^ ^)-(^ ^14^ It is appropriate to emphasize that there are still few studies that have analyzed syphilis trends by Brazilian health regions, which would provide important data for evaluating strategies aimed at addressing the problem in each of these regions.

The objective of this study was to analyze the temporal trend and spatial distribution of acquired syphilis detection rates in the state of Mato Grosso, Brazil, between 2010 and 2021.

## METHODS

**Figure 1:**
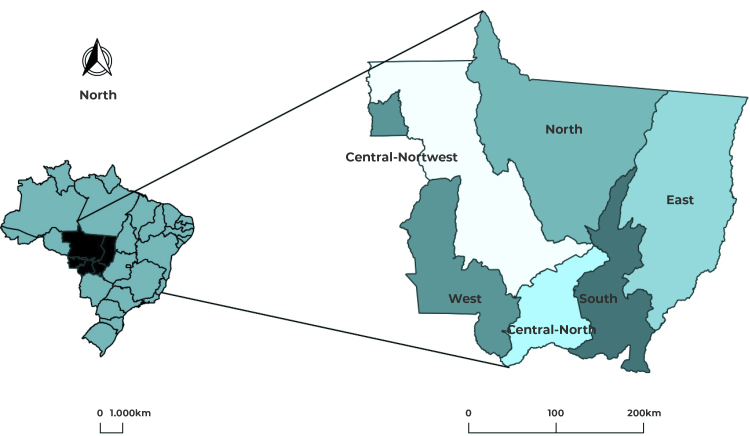
Health macro-regions, Mato Grosso, Brazil, 2021

Source: Cartographic data: IBGE.

This was an ecological study of cases of acquired syphilis, analyzing the temporal trend and describing the spatial distribution of detection rates, by health macro-region, in the state of Mato Grosso. Mato Grosso is located in the Midwest region of Brazil and had an estimated population of 3,658,813 inhabitants in 2022,^15^ in 141 municipalities distributed over six health macro-regions (Figure 1) and 16 regions: North (*Alto Tapajós, Vale do Peixoto, Teles Pires, Vale do Arinos and Norte*), South (*Sul*), East (*Araguaia Xingu, Norte Araguaia Karajá, Médio Araguaia and Garças Araguaia*), West (*Sudoeste* and *Oeste*), Central-North (*Baixada Cuiabana*) and Central-Northwest (*Centro Norte, Médio Norte* and *Noroeste*).

The study population were cases of acquired syphilis reported in the state’s municipalities during the study period, extracted from the SINAN and provided by the Epidemiological Surveillance Coordination (*Coordenadoria de Vigilância Epidemiológica* - COVEPI) of the Mato Grosso State Health Department in November 2022. The population estimates were obtained via TABNET, available on the website of the Brazilian National Health System Information Technology Department (*Departamento de Informática do Sistema Único de Saúde* - DATASUS). The data were organized on an Excel spreadsheet. The detection rate was calculated by dividing the number of reported cases, in the location and period, by the population, in the same location and period, multiplied by 100,000 inhabitants per year.

The detection rates were analyzed according to health macro-region (South, West, North, East, Central-Northwest and Central-North) and three-year periods (2010-2012, 2013-2015, 2016-2018, 2019-2021). In the spatial analyses, the units of analysis were the municipalities of Mato Grosso in the same three-year periods.

Trend analysis of detection rates was carried out, for each health macro-region, using the jointpoint model. The log-transformed detection rates were taken to be dependent variables (y), while the years of the study period were taken to be independent variables (x). The jointpoint model enables trend lines and their inflection points to be adjusted on a logarithmic scale.^16^ ^),(^ ^17^ We calculated annual percentage change (APC) and average annual percentage change (AAPC). Trends were classified as rising (positive APC and p-value < 0.05), falling (negative APC and p-value < 0.05) and stationary (p-value > 0.05). We used the Joinpoint Regression Program, version 4.9.0.0.

When analyzing the distribution of detection rates, we built thematic maps by municipality for each three-year period using the QGIS software, version 3.18, with the SIRGAS 2000 projection and reference system. We used the cartographic database of the state (municipalities, regions and health macro-regions) available on the website of the Brazilian Institute of Geography and Statistics (*Instituto Brasileiro de Geografia e Estatística* - IBGE).^18^

The syphilis detection rates were smoothed by calculating local empirical Bayesian rates [weighted average between the crude rate for the place and the global rate for the region (ratio between the total number of cases and the total population)] using GeoDa software.

The same software was used to build the maps with the local univariate Moran test LISA indicator, in order to analyze the level of local spatial autocorrelation. Quadrant analysis indicates areas of positive spatial association Q1 (++) and Q2 (--), and areas of negative spatial association Q3 (+-) and Q4 (-+). The areas located in quadrants Q1 and Q2 form clusters of acquired syphilis cases of similar values ​​with positive autocorrelation, while the areas located in quadrants Q3 and Q4 do not show similarities between neighboring areas, that is, negative autocorrelation.

The study was approved by the Research Ethics Committee of the *Universidade Federal de Mato Grosso*, as per opinion No. 5.245.07, on February 16, 2022, in accordance with National Health Council Resolution No. 466, dated December 12, 2012. As the study was based only on secondary data, informed consent did not need to be obtained.

## RESULTS

**Figure 2:**
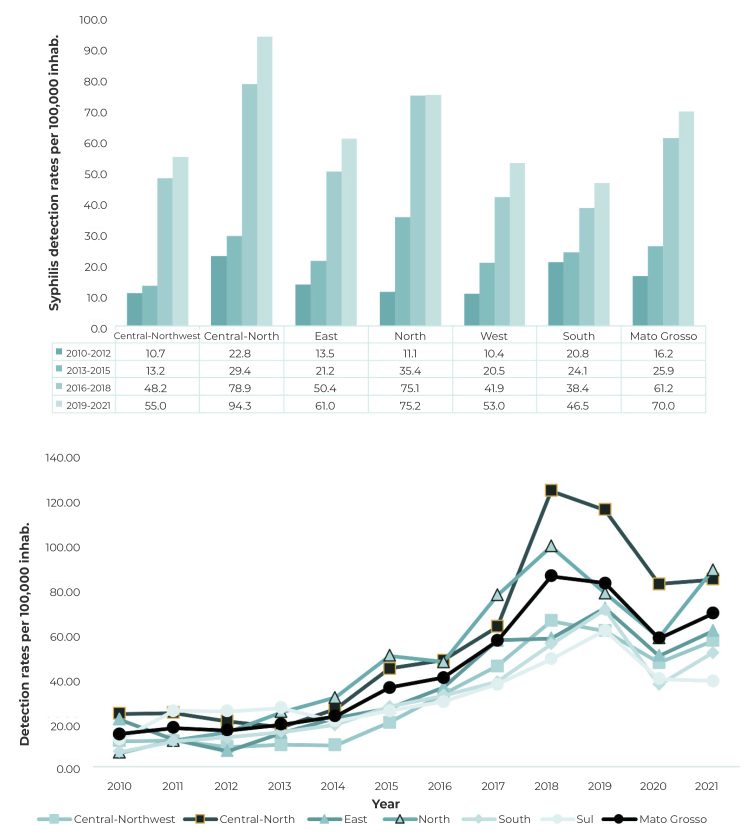
Syphilis detection rates per 100,000 inhabitants by health macro-region, Mato Grosso, Brazil, 2010-2021

We analyzed 17,712 cases of acquired syphilis reported in the state from 2010 to 2021. The detection rate increased during the period, going from 14.7 in 2010, to 69.2 per 100,000 inhabitants in 2021. There was a higher peak of occurrence in 2018 (86.1 per 100,000 inhabitants). Considering the differences in rates between the three-year periods, we found that the acquired syphilis detection rate increased from 16.2 per 100,000 inhabitants, in the first three-year period (2010-2012), to 70 per 100,000 inhabitants in the last three-year period (2019-2021). The rates for the last two three-year periods were higher than the previous three-year periods in all the state’s macro-regions, as shown in Figure 2.

The Central-North macro-region, which includes the *Baixada Cuiabana* health region, where the state’s two largest cities in terms of population are located (the capital, Cuiabá, and Várzea Grande), had higher detection rates in relation to the other macro-regions in almost all periods of the time series, except in the second three-year period, when the Northern macro-region had a higher rate. The increase in detection rates, between the first and the last three-year periods, was more pronounced in the Northern macro-region, where an increase of 677.5% was found, and in the Central-Northwest macro-region, which had an increase of 514%. The Southern macro-region had the lowest increase in rates, namely 223.6%.

In the Central-Northwest macro-region, the highest detection rates in the last two three-year periods (2016-2018 and 2019-2021) occurred in the two largest municipalities in terms of population: Tangará da Serra of the *Médio-Norte* region, (125.3 and 114.5 per 100,000 inhabitants respectively) and Juína in the *Noroeste* region (87.4 and 65.7 per 100,000 inhabitants, respectively), both municipalities being health region headquarters. In the Central-North macro-region, the city of Cuiabá had the highest detection rates in the last two three-year periods (94.2 and 110.1 per 100,000 inhabitants, respectively), followed by Várzea Grande, which had rates of 66 and 89 per 100,000 inhabitants. In the Northern macro-region, Sinop, the largest city in terms of population and headquarters of the *Teles Pires* health region, had the highest acquired syphilis detection rates in the last two three-year periods (140.3 and 122.8 per 100,000 inhabitants, respectively), while Nova Mutum, the fifth largest municipality in that macro-region and part of the same health region, had the second highest rates in the last three-year period: 125.6 and 177.3 per 100,000 inhabitants, respectively.

In the East macro-region, Confresa, the second largest municipality in the *Baixo Araguaia* region, had the highest detection rates in the last two three-year periods (132.2 and 141.8 per 100,000 inhabitants, respectively), followed by Barra do Garças (80.1 and 79.9), the largest municipality in that macro-region and headquarters of the *Garças Araguaia* health region.

The same was found for the West macro-region, where the second largest city in the macro-region, Pontes e Lacerda, in the *Sudoeste* region, had the highest detection rates in the last two three-year periods (83.4 and 125.3, respectively). However, the largest city in that macro-region, Cáceres (*Oeste* region), had much lower detection rates for acquired syphilis (48.9 and 51.7, respectively). Both cities are health region headquarters.

In the Southern macro-region, the municipalities with the highest detection rates were Campo Verde (106.7 and 147.4), the third largest city in the macro-region, and Primavera do Leste, the second largest municipality, with detection rates of 60.5 and 98, although neither of them is a headquarters of the health region.

The joinpoint analysis showed that, in the West, North and East macro-regions, there was a strong trend of rising rates until the middle of the study period (2018-2019), when the curves inflected, and began to show a falling trend (Table 1).

**Table 1:** Temporal trend of acquired syphilis detection rates by macro-health regions, Mato Grosso, Brazil, 2010-2021

Indicators	Period	Trends		Total period	
Macro-regions		APC^a^ (%)	95%CI	AAPC^b^ (%)	95%CI
South	2010-2015	0.1	-15.5;18.6	3.4	-6.0;13.9
	2015-2019	25,9*	7.9;47.0		
	2019-2021	-24.3	-55.6;29.3		
West	2010-2019	27,3*	24.6;30.1	16,4*	11.4;21.7
	2019-2021	-22.1	-40.8;2.6		
North	2010-2018	30,9*	23.0;39.3	17,8*	8.6;29.2
	2018-2021	-11.1	-23.1;2.8		
East	2010-2018	27,7*	14.1;42.8	18,5*	8.6;29.2
	2018-2021	-3.0	-24.0;23.8		
Central-North	2010-2013	-14.4	-56.6;68.5	11.1	-3.5;27.9
	2013-2018	48,2*	29.3;69.8		
	2018-2021	-10.9	-23.8;4.2		
Central-Northwest	2010-2013	-10.3	-42.8;40.4	13,8*	2.1;26.8
	2013-2018	50,2*	26.3;78.6		
	2018-2021	-9.0	-23.6;8.1		
Mato Grosso	2010-2014	12.3	-15.2;48.6	14,5*	5.5;24.2
	2014-2018	39,2*	23.6;56.9		
	2018-2021	-9.5	-19.7;1.9		

a) APC: Annual percentage change; b) AAPC: Average annual percentage change. *p<0,05

The South, Central-North and Central-Northwest macro-regions, as well as the state of Mato Grosso as a whole, showed two inflection points in the trend of acquired syphilis detection rates (the first being positive and the second negative) dividing the series into three periods, with the second period being significant for the four macro-regions, in particular the Central-Northwest and Central-North.

**Figure 3:**
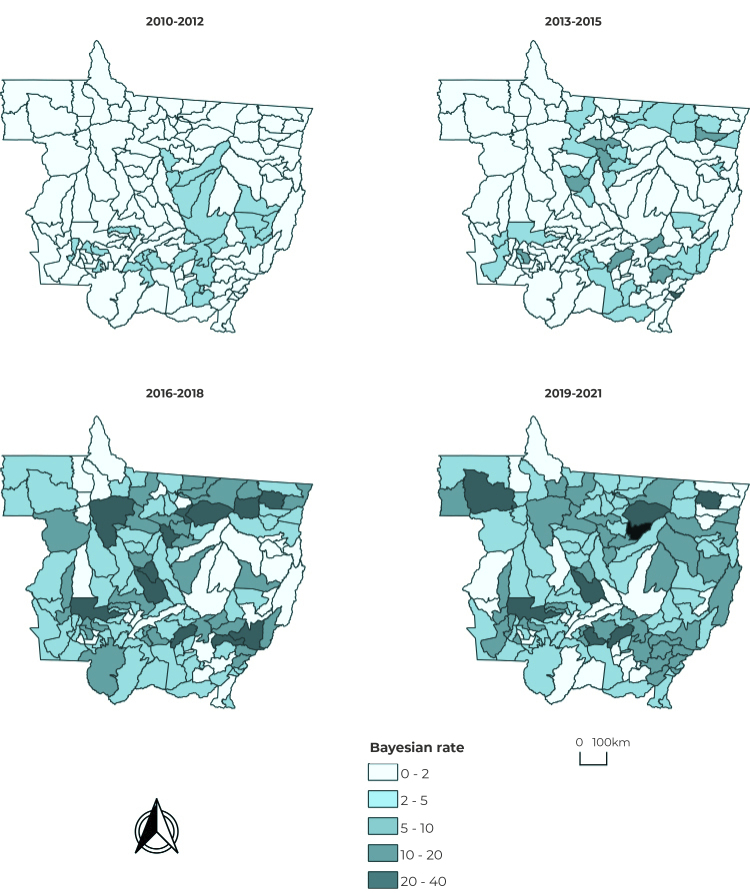
Spatial distribution of the Bayesian syphilis rate per 100,000 inhabitants, in three-year periods, Mato Grosso, Brazil, 2010-2021

Figure 3 presents the spatial distribution of local empirical bayesian rates of acquired syphilis in the municipalities in three-year periods, whereby there was a greater increase in the last two three-year periods in most municipalities.

**Figure 4:**
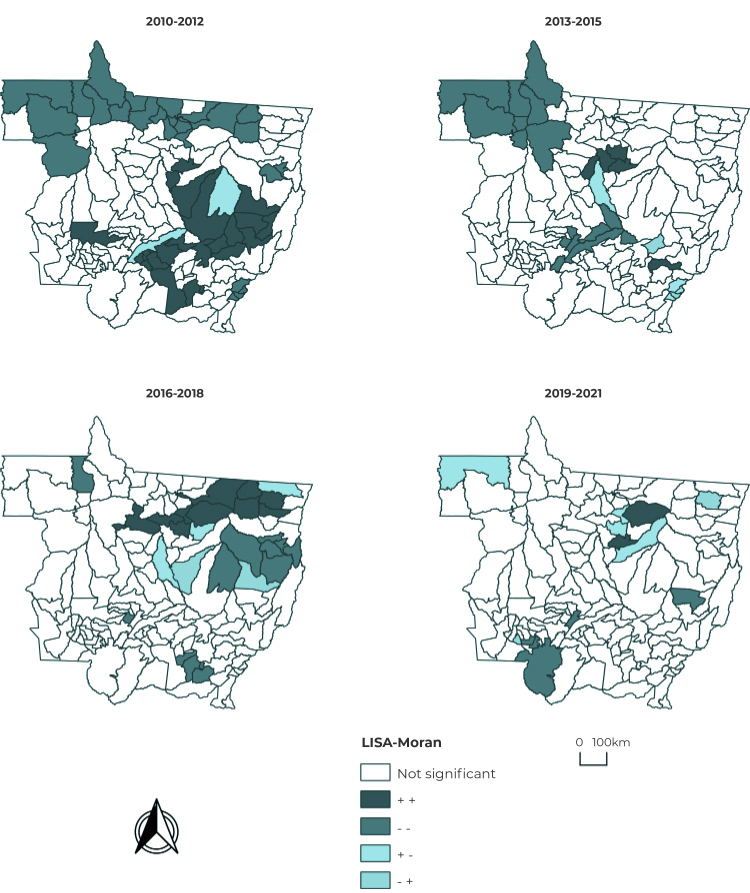
Degree of local (Moran) spatial autocorrelation of the bayesian syphilis rates per 100,000 habitants, Mato Grosso, Brazil, 2010-2012, 2013-2015, 2016-2018, 2019-2021 and 2010-2021

Figure 4 shows the LISA indicator of the univariate local Moran test. Spatial autocorrelation analysis using the local Moran method showed that the largest clusters were found in the Central-Northwest and Northern macro-regions of Mato Grosso, for municipalities in Q2, in the first three-year period. On the other hand, municipalities located in the East and Central-North macro-regions had the largest clusters in Q1. In the second three-year period, there was an increase in the number of municipalities in the cluster in the Central-Northwest macro-region in Q2. The third three-year period showed an increase in the number of municipalities in the Northern macro-region cluster in Q1 and an increase in municipalities in the Eastern macro-region cluster in Q2. In the last three-year period, there was no evidence of an increase in municipalities in any quadrant. The total period was also analyzed, whereby only the Northern macro-region had large clusters in Q1 (Figure 4).

## DISCUSSION

This study showed an increasing trend in acquired syphilis in the state of Mato Grosso, in the period between 2010 and 2021, particularly in the last two three-year periods of the series (2016-2018 and 2019-2021), despite Ministry of Health initiatives to combat the disease with effect from 2016.^1^ ^),(^ ^5^ ^),(^ ^19^

Our analysis of detection rates during the study period showed that the Southern macro-region had the lowest rates, while the Central-North macro-region had the highest rates throughout the time series.

Analysis of the LISA indicator of spatial autocorrelation assessment using local Moran showed an increase in municipalities in the cluster in Q1 and Q2 only in the third three-year period (2016-2018). Other studies have shown an increase in the rate of acquired syphilis in Brazil as a whole^12^ and in other states from 2010 onwards. In São Paulo, between 2011 and 2017 the acquired syphilis rate increased by 225,^13^ while in Paraná the rate increased from 0.7 cases per 100,000 inhabitants in 2010, to 87.5, in 2018.^11^

In 2010, the Ministry of Health included syphilis on the list of diseases for which notification is compulsory,^18^ considering the increase in cases with effect from that year, with positive progression in the detection rate over the years since then, which highlights the epidemic of the disease in the Brazil, reaching its peak in 2019 (51.5 cases per 100,000 inhabitants),^1^ this being a trend also found by this study in Mato Grosso.

The positive increase over the first 10 years of the study’s time series can be attributed to factors such as expansion of testing, especially rapid tests, reduction in the use of condoms by the population, resistance by health professionals to administering penicillin in primary health care services, in addition to the global penicillin shortage, especially between 2014 and 2016, and improvement of notification by the surveillance system.^4^ Despite the improvement in notification, recording is still insufficient to detect all cases of the disease, according to a study that pointed out three basic causes of underreporting: shortcomings in notification and prevention actions, lack of knowledge of the disease on the part of health professionals and the population,^6^ ^),(^ ^11^ as well as failure to fill out notification forms completely, with many fields left blank or filled out as unknown, in addition to important data being missing on the forms.^7^ ^),(^ ^11^

It should be emphasized that in Brazil the definition of syphilis cases only includes active cases of the disease, excluding cases of serological scarring and false positive results, which makes it difficult to compare notification data with other countries, since case definition is different.^13^ ^),(^ ^14^ Another issue of concern refers to failure to distinguish between the primary, secondary, recent latent and other stages (stages that correspond to the acute phase of the disease) on the SINAN notification forms.^13^

In order to prevent the transmission of the disease in different population groups, it is necessary to know the epidemiological profile of syphilis, as well as to develop prevention strategies, which include timely diagnosis and adequate treatment of syphilis, both for the person with syphilis and for their partner, as well as correct use of condoms, in order to ensure that the disease is cured.^8^ ^),(^ ^21^ ^)^ These requirements depend, in part, on the quality of care and require coordination between the different levels of care, so as to contribute to the improvement of lines of care to promote sexual and reproductive health, STI prevention, early syphilis diagnosis, treatment and linkage to follow-up, in order to achieve its control and cure.^8^

In the Brazilian National Health Service, there is an extensive set of primary health care and specialized outpatient services of heterogeneous quality and which are not sufficiently articulated between each other.^21^ ^),(^ ^22^ In Mato Grosso, programmatic vulnerability of STI prevention was assessed in Cuiabá’s Primary Health Care Centers, as per Maison (2014),^23^ and in the Mato Grosso Specialized Outpatient Services.^24^ ^),(^ ^25^

The limitations of this study are inherent to the use of secondary data, given that the information came from cases notified on the SINAN, and therefore underreporting may have occurred, especially during the COVID-19 pandemic. Even so, the findings of this study are essential for identifying priority municipalities for controlling the disease, through action planning. Spatial distribution is also important for municipal surveillance services to monitor cases, with the aim of reducing transmission and controlling the disease.

Despite the limitations mentioned, the results obtained can support acquired syphilis prevention and control strategies in the state of Mato Grosso. The methodology developed in this study can be applied to other locations in order to analyze the acquired syphilis detection rate.

Implementation of comprehensive prevention programs combined with care and access to treatment are important actions for preventing new infections, based on monitoring the actions developed, ranging from promotion of sexual and reproductive health through to the outcome. Coordinated lines of care are also essential for effectively addressing the disease.
